# Recent advance in early oral lesion diagnosis: the application of artificial intelligence-assisted endoscopy

**DOI:** 10.3389/fonc.2025.1686356

**Published:** 2026-01-09

**Authors:** Xinyi Zhao, Hao Lin, Bang Zeng, Renbin Zhou, Lei Ma, Bing Liu, Qiusheng Shan, Tianfu Wu

**Affiliations:** 1 State Key Laboratory of Oral & Maxillofacial Reconstruction and Regeneration, Key Laboratory of Oral Biomedicine Ministry of Education, Hubei Key Laboratory of Stomatology, School & Hospital of Stomatology, Wuhan University, Wuhan, China; 2 Department of Oral & Maxillofacial Head Neck Oncology, School & Hospital of Stomatology, Wuhan University, Wuhan, China

**Keywords:** artificial intelligence, deep learning, endoscopic technology, machine learning, oral cancer

## Abstract

Oral squamous cell carcinoma (OSCC) is a globally prevalent malignancy with high mortality. Early detection is crucial, yet traditional diagnostic methods, including biopsies and imaging techniques like CT and MRI, face limitations in identifying small or superficial lesions. Endoscopic techniques, such as White Light Imaging, Narrow Band Imaging, and Autofluorescence Imaging, enhance visualization of mucosal abnormalities, but their accuracy depends on operator expertise. Recent advancements in artificial intelligence (AI) are transforming endoscopic diagnosis by enabling automated lesion detection, segmentation, and classification through deep learning models like Mask R-CNN and U-Net. These AI-driven approaches improve diagnostic precision, reduce human error, and facilitate early intervention, particularly in resource-limited settings. Challenges persist, including the need for standardized datasets, robust preprocessing methods, and strategies to address overfitting in AI models. Techniques such as transfer learning, data augmentation, and multitask learning are employed to overcome these limitations. AI-assisted endoscopy holds promise for early detection, improved treatment outcomes, and enhanced accessibility, particularly in underserved regions. However, ethical concerns, data privacy, and the necessity for clinical validation remain critical. Future research should prioritize refining AI methodologies and integrating them into clinical workflows to optimize the early diagnosis and management of OSCC, thereby improving patient outcomes and reducing global disease burden.

## 1 Introduction

**Figure 1:**
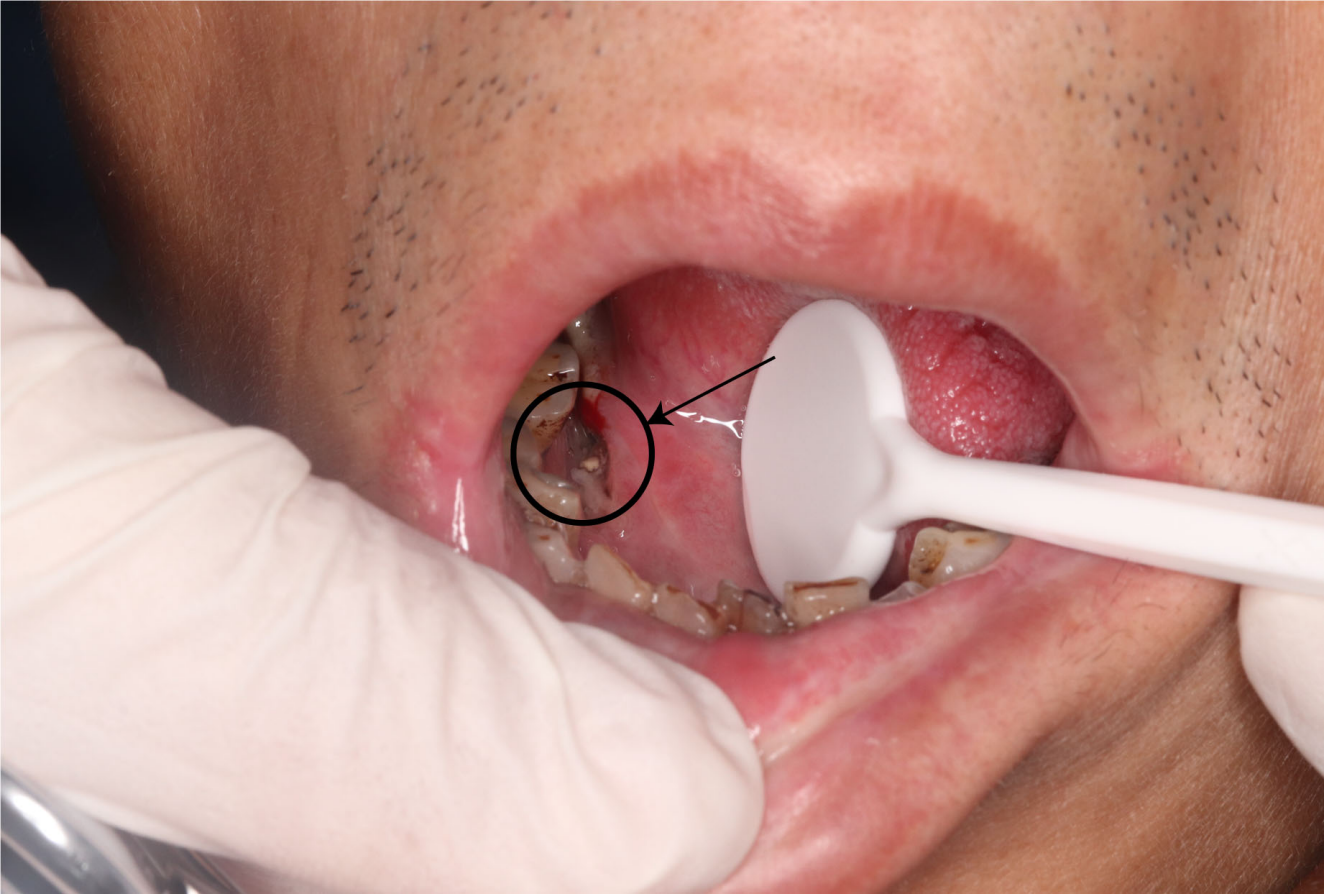
Restricted oral access impedes direct visual diagnosis. As indicated by the black arrow, restricted mouth opening or limited tongue mobility obscures partial visualization of the oral lesion, posing challenges for direct examination. Endoscopic evaluation offers a viable solution to this diagnostic limitation.

Oral squamous cell carcinoma (OSCC) is a common malignancy, ranking sixth globally. In 2022, there were 389,485 new cases and 188,230 deaths, with India having the highest mortality rate (1). Risk factors include smoking, alcohol, smokeless tobacco, and betel nut use (2). These factors increase oral epithelial permeability, raising OSCC risk (3). Genetic mutations also play a role in cancer development. Early symptoms of oral cancer are subtle, often leading to delayed diagnosis. In later stages, it causes severe pain, disfigurement, and functional loss. Early detection of precancerous lesions and timely treatment of OSCC can significantly reduce incidence and mortality (4). Some lesions are located in anatomically hidden areas and may not be easily visualized, as illustrated in **Figure 1**.

Surgical biopsy and histological examination are the gold standard for diagnosing oral cancer, but they are painful, time-consuming, and carry risks like infection and tumor dissemination (5). CT and MRI can detect space-occupying lesions but struggle with small, superficial precancerous lesions, and their resolution is often limited. For oral cancer screening in healthy individuals, clinical exams and biopsies remain essential. Endoscopy, as a real-time, non-invasive tool, aids in detecting benign and malignant oral lesions, including early neoplastic changes (6). Advanced techniques like White Light Imaging (WLI), Narrow Band Imaging (NBI), and autofluorescence improve the detection of abnormal tissues, delineate tumor boundaries, and guide biopsy decisions. Endoscopic assessment is also particularly valuable in patients with advanced OSCC who develop trismus caused by pain, mucosal fibrosis, or radiotherapy, as well as in postoperative reconstruction patients with soft-tissue contracture and limited mouth opening, where conventional intraoral inspection becomes challenging.

Advancements in artificial intelligence (AI) offer innovative solutions to overcome the limitations of endoscopic diagnosis (7). The integration of computer vision and AI has revolutionized healthcare, enhancing disease diagnosis, risk assessment, monitoring, and health policy planning. In the complex anatomical environment of the head and neck, AI systems leverage machine learning (ML) and deep learning (DL) techniques to automatically extract features from endoscopic images for lesion segmentation and classification (8). This assists physicians in performing accurate procedures, improving image quality, and making quicker clinical decisions, ultimately improving diagnostic and therapeutic outcomes (9, 10).

Endoscopy is widely used in endodontics and periodontics but is less commonly applied in the diagnosis of early oral cancer lesions (11–13). Moreover, recent AI-in-dentistry reviews highlight that the key barriers have shifted from algorithm development to clinical integration, including hardware limitations, workflow compatibility, and deployment feasibility in real-world settings. Yet, AI-assisted oral endoscopy and early oral cancer screening remain underrepresented in current literature, with few domain-specific analyses focusing on methodological standards, validation strategies, or practical deployment challenges (14). This gap is particularly evident when contrasted with gastrointestinal and esophageal endoscopy research, where prospective and randomized studies increasingly reshape conclusions regarding AI performance.

Therefore, a focused and comprehensive synthesis is needed. This review discusses traditional endoscopic techniques used for oral lesion diagnosis and explores the potential of AI-assisted endoscopy, focusing on lesion detection, segmentation, and classification. Additionally, it addresses the current challenges—ranging from data harmonization to real-time deployment constraints—and outlines future prospects for AI technologies in this critical area of oral health. The integration of AI with endoscopy enhances early cancer detection, improves treatment outcomes, and helps address specialist shortages, particularly in resource-limited settings.

## 2 Method

A predefined literature search strategy was applied in PubMed using the query “((oral cancer) AND (endoscopic)) AND (artificial intelligence)” to identify studies relevant to AI-assisted oral endoscopy. The search was supplemented by a snowballing approach, in which reference lists of the retrieved articles and related reviews were screened to ensure completeness. Because the available evidence is heterogeneous in study design and outcome reporting, a narrative synthesis was adopted to summarize key themes, including dataset characteristics, image preprocessing techniques, AI model architectures, validation strategies, and diagnostic performance.

## 3 Advances in non-AI-assisted endoscopic techniques

Oral endoscopy is a non-invasive optical technique widely used in head and neck oncology. Modalities such as WLI, NBI, Autofluorescence Imaging (AFI), Raman Spectroscopy (RS), and Confocal Laser Endomicroscopy (CLE) enhance visualization of subtle mucosal abnormalities and allow real-time chairside assessment and high-definition documentation (15). Endoscopists primarily evaluate surface morphology, mucosal coloration, and microvascular architecture, which are closely associated with early dysplasia and carcinogenesis (16). For example, NBI can reveal subtle vascular and surface changes on the soft palate, tongue borders, and buccal mucosa that are often missed during routine examination (17). The principles and limitations of these modalities are summarized in **Table 1**.

**Table 1:** Summarizing the principles and applications of non-artificial intelligence-assisted endoscopic techniques.

Authors&Years	Endoscopic	Principles	Normal tissue presentation	Abnormal tissue presentation	Place	Sensitivity	Specificity	Limitations
Kim DH (18),2020;Zhang X (19),2024;	White light imaging (WLI)	Visual inspection was conducted to observe gross mucosal changes and space-occupying lesions.	Mucous membranes are normal in color, texture and morphology	Ulcers, protuberances, suspicious red and white plaques	Oral cavity, oropharynx	89%	85%	Due to limitations in contrast and resolution, the identification of subtle and occult lesions may be inaccurate, necessitating the integration of other imaging modalities for accurate diagnosis.
Zhou H (20),2018;Muto M (21),2010	Narrow-band imaging (NBI)	The characteristic peaks of the hemoglobin absorption spectrum, in combination with narrow-band optical filters, allow for the enhancement of superficial mucosal vasculature with blue light at 415 nm, while green light at 540 nm enhances abnormal vascular patterns within the submucosal intraepithelial papillary capillary loops (IPCLs).	Brown superficial vessels and cyanotic submucosal vessels	Clearly bordered brown area with scattered brown spots	Larynx, oral cavity, oropharynx, hypopharynx	89%	96%	The optimal approach for surveillance of squamous cell carcinoma (SCC) in the head and neck region remains to be established, as there exists disagreement in the classification criteria for lesions within the oral cavity, oral pharynx, and laryngeal regions.
Moffa A (23),2021;Li J (24),2024;Kim DH (25),2020	Autofluorescence imaging (AFI)	Under blue light or ultraviolet illumination, fluorophores within normal tissue, such as structural proteins, predominantly emit a blue-green fluorescence originating from collagen. Abnormal alterations in tumor cells can result in decreased collagen content or hyperplasia, thereby reducing the fluorescence intensity. Precancerous and malignant lesions exhibit a reddish-purple fluorescence.	Greenish fluorescence	Lack of fluorescence (black spot)	Oral cavity, tongue	100%	93%	Lower specificity and increased difficulty in image interpretation, along with image acquisition biases: characteristics of the light source, hardware limitations in capturing fluorescent signals, and the complexity of the oral environment (including scars and bacteria).

These techniques facilitate early lesion detection and improve biopsy targeting. WLI provides high-resolution visualization of surface structures (18, 19), NBI enhances mucosal microvasculature using wavelength-specific illumination (20, 21), and AFI highlights biochemical alterations associated with premalignant transformation (22–25). Multimodal combinations—such as WLI with NBI, or AFI followed by high-resolution micro-endoscopy—offer complementary diagnostic information and improve lesion localization (26–29).

Reliable interpretation across modalities and devices requires image harmonization. Classical preprocessing approaches such as color constancy and histogram or spectral normalization help mitigate illumination and color variability, while newer techniques employ hyperspectral or software-based transformations to generate NBI-like or spectrum-enhanced images with more consistent mucosal contrast across platforms (30). Artefact handling is equally essential: algorithms can detect and correct specular highlights, and deep-learning models trained on multi-center datasets can identify reflections, bubbles, blood, blur, and instrument artefacts (31, 32). These steps are particularly important in the oral cavity, where saliva, blood, and strong reflections frequently obscure tiny superficial lesions.

The diagnostic value of AFI, although helpful, remains heterogeneous. Handheld devices such as VELscope demonstrate high sensitivity for high-risk lesions but show variable specificity and frequent false positives in inflamed or scarred tissue (33). More recent studies indicate that AF is most reliable as an adjunct for superficial surgical margin assessment, improving delineation of lateral mucosal margins and increasing the likelihood of achieving clear margins (34, 35). However, AF is restricted to the mucosal layer, does not assess deep invasion, and portable AF/DR devices often show less consistent performance than dedicated surgical systems. Thus, AF should be regarded as a complementary tool for margin evaluation and risk stratification rather than a stand-alone diagnostic method.

In summary, while multimodal endoscopy significantly improves visualization and biopsy guidance, variability in image quality, operator dependence, and diagnostic inconsistency remain unresolved barriers. To overcome these limitations, AI-assisted endoscopic analysis has emerged as a powerful complement, enabling automated feature extraction, lesion mapping, and objective risk stratification—paving the way for the next generation of intelligent oral cancer screening tools.

## 4 Advances in AI-assisted endoscopic techniques

Recent advancements in AI for medical image processing have revolutionized endoscopic diagnosis, offering opportunities. AI utilizes advanced algorithms to analyze vast datasets, optimize image quality, and assist in diagnosis. Among these, deep learning techniques, particularly convolutional neural networks (CNNs), have seen rapid development. CNNs, inspired by human neural networks, consist of interconnected layers capable of automatically extracting and analyzing image features without manual input. Their performance is influenced by factors like network depth and hardware capabilities (36, 37). In oral endoscopic diagnosis, AI excels in lesion detection, segmentation, classification, and diagnosis.

### 4.1 Automatic detection and segmentation of lesions

Image segmentation aids in automatic lesion detection by delineating tumor borders and mucosal textures. However, model training requires expert annotation to minimize errors and improve accuracy. **Table 2** outlines key AI models used in automated lesion detection and segmentation.

**Table 2:** General characteristics of AI models incorporated into automated detection and segmentation studies of lesions.

Authors&Years	Clinical validation level	Place	AI model	Datebase	Data type(format)	Purpose of study	Outcomes		External validation or not
Paderno A (38),2023	Retrospective Single-Center	UADT	DL-(MASK R-CNN)-ResNet50	LSCC-653 frames, OSCC-246 frames, and OPSCC-135 frames	NBI image	Combined with NBI for tumor segmentation	DSC	Larynx/Hypopharynx-0.9	No
								oral cavity-0.6	
								oropharynx-0.8	
Paderno A (39),2021	Retrospective Single-Center	OC/OP	DL-FCNN (U-Net 、 U-Net 3 & ResNet)	OSCC-110 frames/OPSCC-116 frames	NBI image	Combined with NBI for OSCC and OPSCC segmentation	DSC	0.76	No
Azam MA (40),2022	Retrospective Multicenter	UADT	DL-SegMENT: based on DeepLabV3+ CNN	LSCC-683 video frames; external verification queues OPSCC and OSCC 116 and 102 images each	Video&image	Combination of WLI and NBI, recognized segmentation LSCC\OSCC\OPSCC	IoU	0.68	Yes
							DSC	0.81	
							Sensitivity	0.95	
							Precision	0.78	
							Accuracy	0.97	
Sampieri C (41),2024	Retrospective Multicenter	Larynx	DL-(SegMENT-Plus)	LSCC-557 WL&NBI images	WLI&NBI image	Combined with WLI and NBI for UATD cancer segmentation	IoU	0.68	Yes
							DSC	0.81	
							Accuracy	0.97	
							Time	25.6 frames per second	

AI, artificial intelligence; DL, deep learning; UADT, upper aerodigestive tract; OSCC, oral squamous cell carcinoma; OPSCC, oropharyngeal squamous cell carcinoma; LSCC, Larynx squamous cell carcinoma; WLI, white light imaging; NBI, narrow-band imaging; IoU, intersection over union; DSC, dice similarity coefficient.

Paderno et al. developed Mask R-CNN for NBI endoscopic tumor segmentation, achieving success in upper aerodigestive tract (UADT) but facing challenges in the oral cavity due to mucosal diversity and confounding factors like teeth and dentures (38).In another study, U-Net3 outperformed other FCNNs, demonstrating fast training and promising diagnostic accuracy for oral and oropharyngeal NBI videos (39).Azam et al. introduced SegMENT, a DeepLabV3+ model optimized with Xception, which excelled in early OSCC and OPSCC detection, precise biopsies, and margin selection (40). Sampieri et al. improved SegMENT with SegMENT-Plus, achieving better segmentation accuracy, though complex cases like overlapping lesions remain challenging (41).

AI-driven automated lesion detection and segmentation enhance diagnostic accuracy in anatomically complex oral regions while reducing human error. This approach enables faster and more precise tumor identification, providing a reliable foundation for subsequent classification and facilitating targeted clinical interventions.

### 4.2 Determination and classification of lesion types

Oral malignancies, especially OSCC, can appear as ulcerative, erosive, or nodular lesions. AI frameworks typically start by identifying basic mucosal lesions, which helps distinguish high- and low-risk lesions. Gomes et al. developed a deep-learning model using ResNet-50, VGG16, InceptionV3, and Xception, with InceptionV3 selected for hyperparameter optimization. The model achieved over 70% accuracy in classifying six lesion categories. **Table 3** outlines key AI models for lesion detection and classification (42).

**Table 3:** General characteristics of AI models included in lesion type determination and classification studies.

Authors&Years	Clinical validation level	Place	AI model	Database	Data type(format)	Purpose of study	Outcomes		External validation or not
Gomes RFT (42),2023	Retrospective Multicenter	Oral cavity	DL-CNN- InceptionV3	5069 images	Image	Classification of six primary lesions	Sensitivity	0.85	Yes
							Specificity	0.97	
							Precision	0.86	
							F1 score	0.85	
Fu Q (43),2020	Retrospective Multicenter	Oral cavity	DL-DenseNet121	Development dataset-1469; Internal validation dataset-401; External validation dataset-420	Image	Distinguishing OSCC from Non-OSCC Oral Diseases	Accuracy	0.92	Yes
							Sensitivity	0.91	
							Specificity	0.94	
Tanriver G (44),2021	Retrospective Single-Center (with Public Data)	Oral cavity	DL-Mask R-CNN	652 photographic images of oral lesions	Image	Classification Benign, OPMD, Cancer	DSC	0.93	No
Ye Y (45),2024	Retrospective Multicenter	Oral cavity	DL-CNN-YOLOX	1419 photographs. OLP-262, OLK-328, OSCC-799, Normal-30.	Image	Detection of multiple oral lesions including OLP, OLK and OSCC	Sensitivity	0.85	Yes
							Specificity	0.95	
							Precision	0.87	
Inaba A (47),2020	Retrospective Single-Center	Laryngopharynx	DL-RetinaNet: ResNet-50	SLPC-400; normal-800	NBI image	Diagnosis of superficial laryngeal cancer	Sensitivity	0.96	No
							Specificity	0.98	Yes
Heo J (48),2022	Retrospective Multicenter	Tongue	DL-CNN-DensetNet169	Malignant lesions-1941; nonmalignant lesions-3635 nonmalignant	NBI image	Diagnosing Tongue Cancer	Accuracy	0.85	
							Sensitivity	0.81	
							Specificity	0.87	
Talwar V (49),2023	Retrospective Multicenter	Oral cavity	DL-CNN-DensetNet201	Composed of 1120 suspicious and 1058 non-suspicious oral photography images	Image	Classification of suspicious lesions	Sensitivity	0.85	Yes
							Specificity	0.83	
							Precision	0.86	
							F1 score	0.86	
Islam MM (60),2023	Observational Public Database	Oral cavity	DL-DeIT、VGG19,MobileNet	Benign-165; malignant-158.Enhancement techniques-1,320 training images of benign lesions and 1,273 training images of malignant lesions	Image	Recognizing benign and malignant oral lesions	Accuracy	VGG19-1	No
							Sensitivity	MobileNet-1	
							Specificity	DeIT-0.99	
Bansal K (65),2022	Retrospective Study	Oral cancer (lips and tongues)	DL/DTL-ResNet5, MobileNetV2,VGG19,VGG16,DenseNet	Cancer-87; non-cancer-44	Histopathology Dataset + Real-time Images	Diagnosing Oral Cancer	Accuracy Sensitivity Specificity	0.96	No
								0.98	
								0.94	

AI, artificial intelligence; DL, deep learning; UADT, upper aerodigestive tract; NBI, narrow-band imaging; AFI, autofluorescence imaging; OPMD, oral potentially malignant disorders; OSCC, oral squamous cell carcinoma; OLP, oral lichen planus; OLK, oral leukoplakia; DSC, dice similarity coefficient.

Smartphone photography has become an essential tool for early diagnosis, especially in regions with limited medical expertise. Fu’s team developed a deep-learning algorithm using intraoral images, achieving an AUC of 0.995 in detecting early OSCC with high sensitivity and specificity, outperforming human experts (43). Tanriver et al. proposed a two-stage model to detect and classify lesions, utilizing U-Net and Mask R-CNN with ResNet backbones, showing promising results for oral cancer screening (44). Similarly, Ye et al. developed “Oral-Tec,” an Android app utilizing YOLOX for detecting oral lesions, enhancing accessibility in community hospitals (45, 46).

Inaba et al. used the RetinaNet model to diagnose superficial pharyngeal and laryngeal cancers, achieving a sensitivity of 95.5% (47). Heo’s team trained a model on 5,576 endoscopic images to identify tongue cancer, with DenseNet169 yielding the best results (48). Talwar et al. used DenseNet201 to classify smartphone images of oral potentially malignant disorders (OPMD), emphasizing the trade-offs between performance, speed, and network size in algorithm selection (49).

Accurate lesion classification is critical for assessing malignancy risk and guiding treatment decisions. AI models that categorize lesions as benign, premalignant, or malignant enable timely intervention, improving risk stratification and personalized management—key factors in reducing morbidity and mortality. Smartphone-based algorithms further enhance accessibility to diagnostic tools in resource-limited settings, facilitating rapid screening and early detection, which may improve survival rates through prompt treatment.

### 4.3 Assessment and evaluation of lesion depth

In oncology, lesion type is crucial for predicting patient prognosis, with local depth of invasion (DOI) serving as a key factor in treatment decisions. Accurate assessment of invasion margins is essential for personalized management. For gastrointestinal tumors, DOI classification based on macroscopic endoscopic morphology and pathology has shown promise when combined with deep learning (50). Expanding on this, Tateya et al. demonstrated that NBI can predict DOI in superficial oral cancer (51). Furthermore, Yumii et al. applied machine learning to classify NBI images of squamous cell carcinoma and *in situ* lesions, using five-fold cross-validation to confirm its diagnostic utility for subepithelial DOI, thereby reducing the risk of over-resection and its associated complications, such as postoperative dysphagia and scar adhesion (52).

Accurate DOI assessment is essential for treatment planning. AI-based endoscopic DOI classification optimizes surgical strategy, balancing tumor resection with functional preservation while reducing overtreatment and improving outcomes (53).

## 5 Technical challenges and responses

Building on the advantages of endoscope-assisted AI in diagnosing early oral lesions, the development of AI-driven diagnostic tools for oral cancer faces several significant technical challenges. These include the need for standardized datasets, refined preprocessing techniques, and the establishment of robust model training strategies. Overcoming these obstacles is crucial to enhancing the diagnostic accuracy and applicability of AI in clinical settings.

### 5.1 Standardization of datasets

Training AI models typically involves dividing data into training, validation, and test sets in an 8:1:1 ratio (44). The training set is used to optimize network parameters over multiple epochs, the validation set evaluates model performance during training, and the test set assesses final performance (54). Dataset quality, size, and format critically impact model outcomes.

#### 5.1.1 Quality of datasets

Missed tumor detection often results from hard-to-identify lesions or poor mucosal observation. With advances in smartphone cameras, clinicians can easily capture high-definition intraoral images. However, concealed areas like the posterior tongue or soft palate require endoscopic imaging for comprehensive observation. AI systems now assist in real-time endoscope withdrawal to ensure full mucosal examination (55).

**Figure 2:**
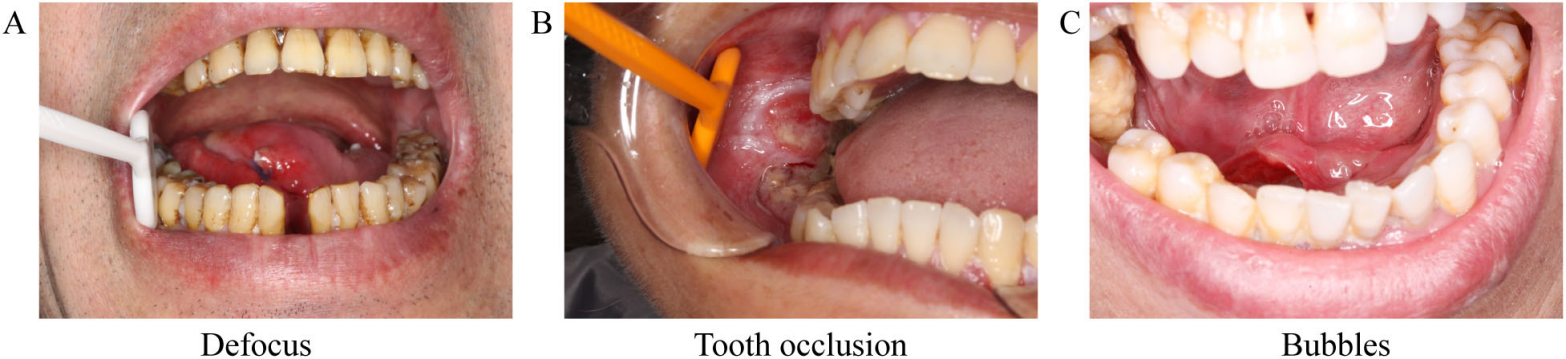
Common quality problems with endoscopic images that often interfere with artificial intelligence models to make diagnoses. **(A)** due to defocus; **(B)** due to tooth occlusion; **(C)** due to bubbles occlusion.

Training datasets must exclude low-quality images, such as duplicates, blurry photos, or those obscured by biological materials (e.g., mucus or debris). During the training of AI models, it is crucial to exclude images of poor quality, as exemplified in **Figure 2**. Ali et al. developed an AI program to detect and restore endoscopic artifacts like motion blur, bubbles, and pixel saturation, enhancing image analysis performance (56).

#### 5.1.2 Scale of datasets

**Figure 3:**
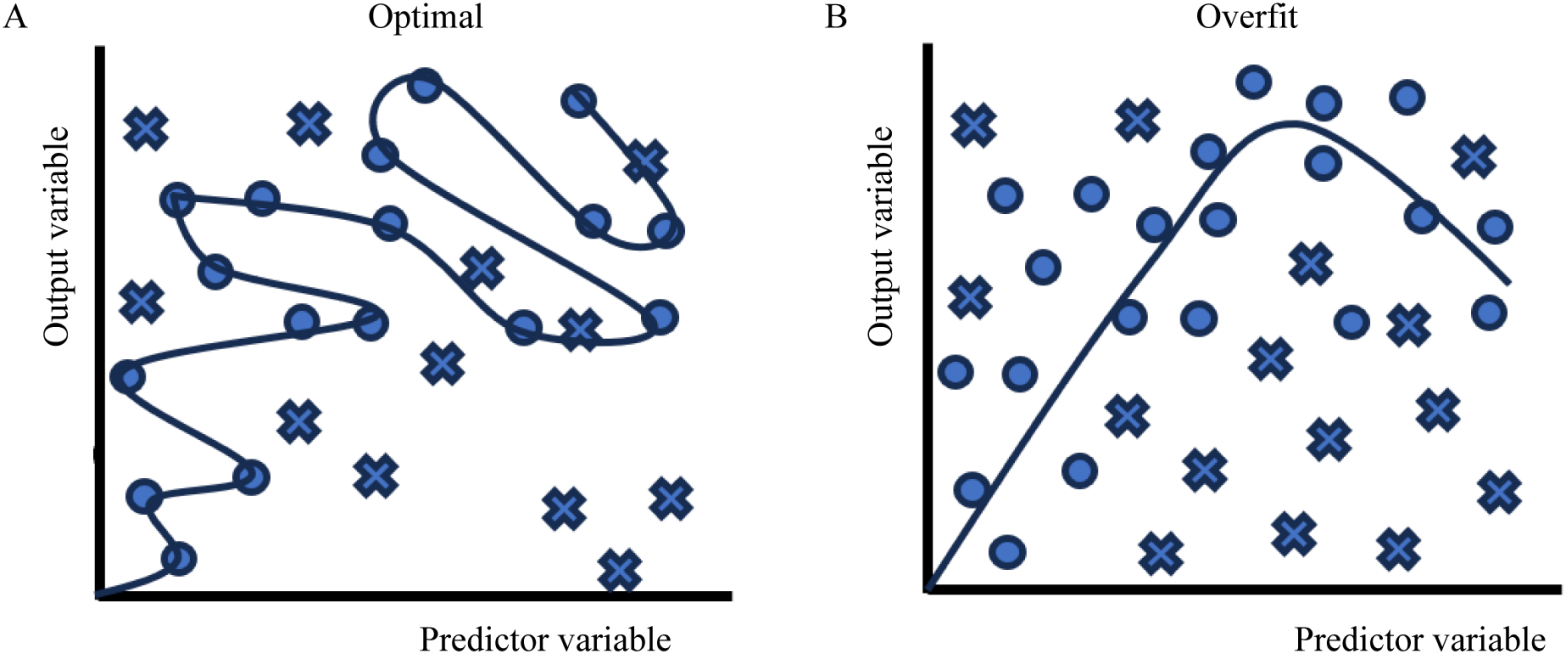
Comparison of model fitting behaviors. **(A)** Optimal fitting demonstrates appropriate pattern capture with smooth curves, while **(B)** overfitting shows excessive adherence to training data points (blue circles/crosses), resulting in loss of generalization capability. Predictor-output relationships are plotted on normalized axes.

AI encompasses both ML and DL methodologies. ML utilizes labeled data for pattern recognition without explicit programming, whereas DL employs multilayer neural networks to extract features from large datasets autonomously. Although DL demonstrates superior performance in image and speech recognition tasks, it demands substantial computational resources and training data (57). In contrast, ML algorithms remain more interpretable and efficient for smaller-scale applications, maintaining robust performance in targeted problem-solving (5). Deep learning, which outperforms traditional machine learning in image recognition, requires large datasets and computational resources. Smaller datasets risk overfitting in complex neural networks, reducing accuracy and generalization (54). In oral cancer diagnosis, the limited availability of endoscopic and photographic images, coupled with quality screening, restricts the dataset size. Techniques like data augmentation, transfer learning, and multitask learning help improve performance and prevent overfitting. The overfitting phenomenon is schematically illustrated in **Figure 3**, as excessive complexity leads to poor generalization performance.

#### 5.1.3 Format of datasets

Dynamic video data provides richer diagnostic information, such as lesion location and size, and achieves higher accuracy than static images (58). However, videos require more storage and longer interpretation times. Additionally, variations in resolution across imaging devices may affect diagnostic consistency, though further research is needed. Meanwhile, well-structured, high-quality datasets reduce diagnostic biases and errors in AI models (42).

### 5.2 Preprocessing of endoscopic images

Preprocessing is a key step in AI image analysis, transforming subjective tasks into quantifiable processes to extract relevant information. It involves two main aspects: extracting valid segments and optimizing image quality, ensuring regions of interest are preserved while eliminating confounding factors.

#### 5.2.1 Extraction of valid segments: attention mechanism

Endoscopic videos often span multiple anatomical sites, leading to visual fatigue and reduced diagnostic accuracy. Efficiently extracting valuable images simplifies subsequent deep learning tasks, enhances computational efficiency, and optimizes storage (59).

The attention mechanism focuses processing resources on critical image areas. Song et al. leveraged Vision Transformers (ViT) and Swin Transformers for oral cancer image classification. ViT uses shifted window attention to capture hierarchical image structures, while Swin Transformers divide images into patches, dynamically focusing on key areas. Both outperformed traditional CNNs like VGG19 and ResNet50 in accuracy (60). Swin Transformers also excelled in detecting suspicious oral lesions using white-light images (49).

Additionally, the Guided Attention Inference Network (GAIN) employs attention maps from weakly supervised networks to improve classification and segmentation. Figueroa et al. used data augmentation and GAIN’s two-stage training to enhance CNN accuracy, generating precise segmentation maps for mobile screening devices (61).

#### 5.2.2 Optimization of image quality - image augmentation

**Figure 4:**
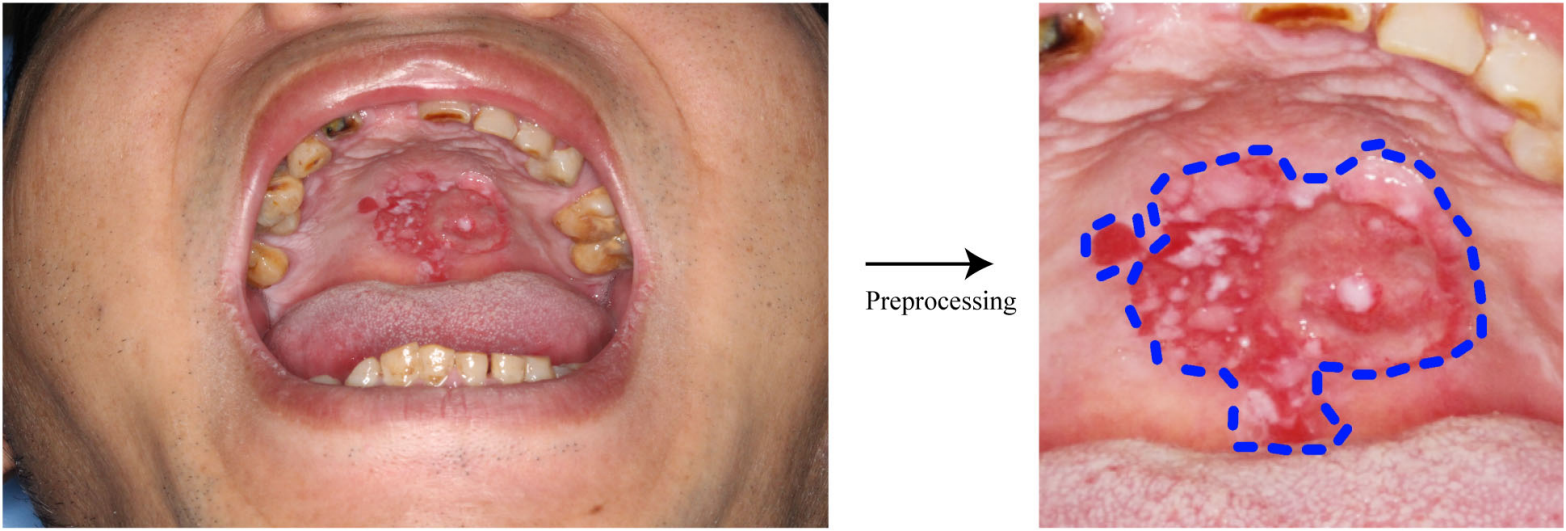
Preprocessing of pictures. Images obtained directly by endoscopy are preprocessed by changing contrast, brightness, cropping, etc., and are more easily learned by AI models.

Variations in equipment, lighting, and imaging angles can degrade endoscopic image quality, complicating AI’s ability to differentiate subtle features. Image augmentation techniques address these challenges by applying transformations like cropping, rotation, brightness adjustments, and histogram equalization to enhance diagnostic performance (57, 58), as illustrated in **Figure 4**. For example, random contrast changes (0.8–3) or rotations (-20° to 20°) generate diverse training samples, mitigating overfitting in small datasets (38).

When basic augmentation is insufficient, deformable techniques simulate clinical variations like tissue deformation or artifacts, increasing sample diversity. Methods such as random displacement fields and deformable image registration have shown promise, particularly in standardized imaging like CT and MRI (54). Shamim et al. found augmented models performed better than those trained on limited datasets (63). By enhancing texture information and standardizing data, preprocessing with augmentation techniques lays the foundation for accurate lesion segmentation and improved diagnostic efficiency (64).

### 5.3 Training and overfitting: transfer learning

Data augmentation can expand training datasets, but is insufficient to fully address overfitting. Transfer learning offers a solution by leveraging pre-trained models for new tasks, reducing training time, computational demands, and the risk of overfitting.

Fu et al. addressed the lack of OSCC data by using ImageNet pre-trained models, freezing convolutional layer weights, and retraining fully connected layers, improving efficiency (43). Similarly, Shamim et al. applied transfer learning to various models (e.g., AlexNet, ResNet50, and Inceptionv3) for automated oral lesion pre-screening (62). Islam et al. evaluated three transfer learning models—DeIT, VGG19, and MobileNet—achieving 100% accuracy with VGG19 and MobileNet for benign and malignant lesion classification (61). Marzouk et al. combined transfer learning with hybrid optimizers (e.g., ADAM and SGD), achieving 92.41% accuracy in real-time datasets, though dataset limitations hindered generalization (65). Bansal et al. validated DenseNet-169-based AIDTL-OCCM for effective oral cancer detection on lips and tongue (66).

Multitask learning complements transfer learning by improving generalization through shared knowledge across related tasks. For example, Fu et al. implemented a multitask loss combining camera type classification with OSCC detection, enhancing feature extraction (43). Li et al. developed MTN-ResNet50 for tumor staging, lymph node staging, and histological grading, showing no overfitting during validation (67). The team also designed MTN variants (e.g., MTN-AlexNet, MTN-Transformer), which outperformed single-task models (68).

Regularization techniques like dropout and weight decay further mitigate overfitting. Dropout randomly deactivates neurons during training, reducing dependency on specific neurons (69). Weight decay penalizes large weights, constraining the model for better generalization (43). By combining transfer learning, multitask learning, and regularization, models achieve better performance with limited datasets while reducing overfitting risks.

Overcoming these technical challenges through advances in transfer learning, image augmentation, and dataset standardization will enhance AI-assisted diagnostic performance in oral oncology. Continued refinement of these methodologies promises to improve clinical applicability and facilitate integration into routine screening protocols.

### 5.4 Real-world deployment constraints: latency, throughput, and on-device feasibility

Deploying AI-assisted oral endoscopy in resource-limited or portable settings requires explicit consideration of latency, throughput, and on-device feasibility. To support real-time screening, inference must be fast enough to overlay predictions on the live endoscopic stream without interrupting the examination (70). Lightweight or quantized models are therefore necessary, as continuous high-resolution processing on smartphones or handheld endoscopes imposes constraints on computation, heat, battery consumption, and network reliability. Cloud-based inference is often impractical due to bandwidth limitations and privacy concerns, making hybrid on-device strategies preferable. User interfaces must also remain intuitive, displaying risk maps or alerts without obscuring anatomical detail. Recent smartphone-based dental and tele-dentistry studies similarly identify latency, device capability, and workflow integration as the primary barriers to real-world deployment (14).

## 6 Multimodal integration of endoscopic and histopathologic data

While endoscopic AI models show promising performance in detecting early oral lesions, their clinical utility remains limited by the lack of integration with histopathology, the diagnostic gold standard. Endoscopy captures macroscopic mucosal features such as color, surface texture, and vascular alterations, whereas histopathology provides microscopic confirmation of dysplasia and invasion. Establishing meaningful links between these modalities is essential for AI systems aiming to infer histologic severity directly from endoscopic views.

Several structural and technical barriers impede such multimodal integration. First, clinical pipelines for endoscopy and pathology are largely siloed. Even fully digital pathology laboratories store WSIs, endoscopic images, and clinical metadata in separate and often incompatible systems, hindering large-scale multimodal dataset construction (71). Regional digital pathology networks face additional challenges—including scanner heterogeneity, diverse LIS infrastructures, and lack of unified data standards—which further obstruct cross-modality data linkage (72).

Second, accurate spatial correspondence between endoscopic fields and histologic sections is rarely achievable, as biopsy orientation varies and tissues undergo deformation during fixation, embedding, and sectioning. As a result, WSIs no longer precisely represent the region visualized endoscopically (73). This mismatch makes supervised pixel-level multimodal learning largely infeasible and explains why most current AI models remain single-modality. A recent systematic review of AI in digital histopathology similarly highlights that multimodal integration with endoscopic imaging is seldom realized due to the absence of paired datasets and workflow limitations (74).However, recent studies have integrated endoscopic hyperspectral imaging (HSI) with deep learning, producing the first large-scale *in vivo* annotated oral HSI dataset and demonstrating that architectures such as DeepLabv3 and U-Net can reliably differentiate intraoral tissue types with high F1-scores, highlighting the potential of this approach for noninvasive pathological assessment and early cancer detection (75).

Despite these barriers, emerging approaches offer potential solutions. Contrastive learning, cross-attention architectures, and weakly supervised co-localization may allow AI models to infer cross-modality relationships without exact spatial alignment. At the system level, the adoption of DICOM-WSI formats, vendor-neutral archives, and standardized digital pathology workflows may eventually facilitate the development of integrated endoscopy–pathology datasets. These advances will be essential for creating AI systems capable of linking endoscopic phenotypes with histologic truth, supporting targeted biopsy, and improving early cancer risk stratification.

## 7 Future directions

AI, combined with endoscopic imaging, holds promise for early head and neck tumor detection, offering precise screening and personalized prevention strategies.

### 7.1 Early detection and patient engagement

AI-driven diagnostics, particularly in high-risk patients, enhance the efficiency of early detection and treatment, thereby improving patient prognosis. Additionally, AI aids in locating occult cancers, guiding biopsies, and optimizing treatment plans. By providing relatively objective diagnostic results, AI facilitates informed consent, thereby enhancing patients’ understanding and trust in their treatment plans.

### 7.2 AI ethical and privacy concerns

AI reliability is a key concern, particularly with large language models, as their outputs must be evaluated for accuracy, sensitivity, and reproducibility. Additionally, data quality significantly influences AI performance, with proper preprocessing allowing models to perform well even with limited datasets. Ethical oversight and privacy protections are also essential to ensure the responsible use of AI in healthcare, safeguarding patient rights and maintaining trust in these technologies.

### 7.3 AI in clinical practice

AI-endoscopy accelerates training for non-specialists, promoting equitable healthcare, particularly in resource-limited settings. Although Chang et al. utilized tools like ChatGPT-4 and patient data to generate follow-up recommendations aligned with USMSTF guidelines, the reproducibility and reliability of these tools remain unclear (76). However, AI should remain a supportive tool, not a replacement for clinical decision-making or pathology. AI-endoscopy integration offers great potential for improving healthcare, but it should complement human expertise, not replace it, especially in diagnosing and treating oral cancer.

## 8 Conclusion

This review aims to improve early oral cancer diagnosis accuracy and enhance patient quality of life by outlining the principles, advantages, and limitations of endoscopic techniques for detecting lesions in concealed oral areas. The integration of artificial intelligence with endoscopy has shown great promise in oral cancer lesion detection and classification, facilitating precise and rapid early diagnosis by non-specialists and providing reliable diagnostic options for underserved regions with limited medical resources. However, challenges like dataset quality and overfitting persist, requiring strategies such as data augmentation and transfer learning. Future efforts should focus on advancing research and technology to better serve oral cancer patients and optimize early diagnosis and treatment.
